# Mutations in *APC*, *CTNNB1 *and *K-ras *genes and expression of hMLH1 in sporadic colorectal carcinomas from the Netherlands Cohort Study

**DOI:** 10.1186/1471-2407-5-160

**Published:** 2005-12-15

**Authors:** Margreet Lüchtenborg, Matty P Weijenberg, Petra A Wark, A Merdan Saritas, Guido MJM Roemen, Goos NP van Muijen, Adriaan P de Bruïne, Piet A van den Brandt, Anton FPM de Goeij

**Affiliations:** 1 Nutrition and Toxicology Research Institute Maastricht (NUTRIM), Department of Epidemiology, Maastricht University, Maastricht, The Netherlands; 2 Division of Human Nutrition, Wageningen University, Wageningen, the Netherlands; 3 Nutrition and Toxicology Research Institute Maastricht (NUTRIM), Department of Pathology, Maastricht University, Maastricht, The Netherlands; 4 Department of Pathology, University Medical Centre St. Radboud, Nijmegen, The Netherlands; 5 Research Institute Growth and Development (GROW), Department of Pathology, Maastricht University, Maastricht, The Netherlands

## Abstract

**Background:**

The early to intermediate stages of the majority of colorectal tumours are thought to be driven by aberrations in the Wnt (*APC*, *CTNNB1*) and Ras (*K-ras*) pathways. A smaller proportion of cancers shows mismatch repair deficiency. The aim of this study was to analyse the co-occurrence of these genetic alterations in relation to tumour and patient characteristics.

**Methods:**

In a group of 656 unselected sporadic colorectal cancer patients, aberrations in the *APC*, *K-ras*, *CTNNB1 *genes, and expression of hMLH1 were investigated. Additionally, tumours were divided in groups based on molecular features and compared with respect to patient's age at diagnosis, sex, family history of colorectal cancer, tumour sub-localisation, Dukes' stage and differentiation.

**Results:**

Mutations at the phosphorylation sites (codons 31, 33, 37, and 45) in the *CTNNB1 *gene were observed in tumours from only 5/464 patients. Tumours with truncating *APC *mutations and activating *K-ras *mutations in codons 12 and 13 occurred at similar frequencies (37% (245/656) and 36% (235/656), respectively). Seventeen percent of tumours harboured both an *APC *and a *K-ras *mutation (109/656). Nine percent of all tumours (58/656) lacked hMLH1 expression. Patients harbouring a tumour with absent hMLH1 expression were older, more often women, more often had proximal colon tumours that showed poorer differentiation when compared to patients harbouring tumours with an *APC *and/or *K-ras *mutation.

**Conclusion:**

*CTNNB1 *mutations seem to be of minor importance in sporadic colorectal cancer. The main differences in tumour and patient characteristics are found between groups of patients based on mismatch repair deficiency.

## Background

Carcinogenesis is a multi-step process that involves the accumulation of numerous genetic and epigenetic changes in cells [1]. These genetic aberrations affect a limited number of identified pathways that are involved in (colorectal) carcinogenesis [2]. A genetic model for sporadic colorectal cancer has been proposed, which describes the sequential accumulation of specific genetic alterations in various pathways, involving tumour suppressor genes (e.g. *APC*, *SMAD4*, *TP53*) and oncogenes (e.g. *CTNNB1*, *K-ras*) [3,4]. Important molecular pathways that upon activation affect the early and intermediate stages of colorectal carcinogenesis are the Wnt and Ras signalling pathways, whereas *TP53 *inactivation is considered a late event.

Activation of the Wnt pathway plays a central role in the aetiology of most colorectal cancers and is often the result of mutations in the N-terminal domain of the *APC *gene, that lead to partial or complete loss of this region and thereby to loss of the β-catenin regulating function [5,6]. Conversely, in tumours lacking these *APC *mutations [7], activating missense mutations at one of the phosphorylation sites at codons 31, 33, 37 and 45 of exon 3 of the *CTNNB1 *gene (encoding the β-catenin protein) can render it stable as it can no longer be tagged for cellular degradation. Activation of the Ras pathway in cancer is marked by the loss of the intrinsic GTPase activity of the Ras protein, which can be ascribed to missense mutations in codons 12 and 13 of exon 1, which are responsible for 90% activating mutations in the of the *K-ras *gene [8].

According to the paradigm for colorectal cancer development, mutations in the *APC *and *K-ras *are thought to contribute to the early developmental stages of colorectal cancer [3]. However, a recent study based on the analysis of *APC*, *K-ras *and *TP53 *genes concluded that simultaneous occurrence of all three genetic alterations is rare and that multiple genetic pathways may be relevant to colorectal cancer [9].

Genetic instability is seen in most types of cancer [10]. Two distinct types of genetic instability appear to occur in colorectal cancer [11]: chromosomal and microsatellite instability. Chromosomal instability results in gains or losses of entire chromosomes or parts of them, and gives rise to aneuploid tumours and occurs in the majority of cancers. A smaller proportion of colorectal cancers displays microsatellite instability, represented by diploid cells acquiring high mutation rates, and was found to be associated with defective mismatch repair [12]. These tumours are less likely to harbour mutations in genes associated with chromosomally instable and generally aneuploid tumours, such as *APC*, *K-ras *and *TP5*3 [13-21], suggesting that these tumours form a distinct group. Moreover, microsatellite instable tumours are found predominantly in the proximal colon [22,23], are more likely to occur in patients with a positive family history of colorectal cancer [22,23], are often less differentiated than microsatellite stable tumours [22], and occur more frequently in women [24] and at older age [25]. Moreover, in tumours displaying microsatellite instability, mutations the *CTNNB1 *gene were more frequent [26].

Considering the aforementioned issues, there is a need for studies addressing the heterogeneity of affected genes involved in early to intermediate colorectal cancer development in large groups of patients. We have previously studied the occurrence of *APC *and *K-ras *mutations separately [27,28]. In the current study, in addition to investigating mutations in the *APC*, *CTNNB1 *and *K-ras *genes as well as mismatch repair deficiency by means of hMLH1 expression, and combinations of these aberrations, their relation with various tumour and patient characteristics were studied in a large, unselected group of incident colorectal cancer patients.

## Methods

### Study population

The prospective Netherlands Cohort Study on diet and cancer was initiated in September 1986. The study design has been described in detail elsewhere [29]. The study population originated from 204 municipal population registries throughout the Netherlands, and included a total of 58,279 men and 62,573 women between the ages of 55 and 69 years at baseline.

Incident cancer cases are identified by monitoring of the entire cohort for cancer occurrence through annual record linkage to the Netherlands Cancer Registry, i.e. nine regional cancer registries throughout the Netherlands, and to PALGA, a nationwide network and registry of histo- and cytopathology [30]. Together, the NCR and PALGA provide a near 100% coverage of the municipalities included in the NLCS. The first 2.3 years of follow up were excluded because of possible pre-clinical disease affecting exposure status and because of incomplete nationwide coverage of PALGA in some of the municipalities included in the NLCS in that period. From 1989 until 1994, 929 incident cases with histologically confirmed colorectal cancer were identified within the cohort, of whom 819 could also be linked to a PALGA report of the lesion.

The PALGA reports were used to identify and locate tumour tissue from eligible colorectal cancer patients in Dutch pathology laboratories. Colon and rectal cancer were classified according to site as follows, colon: cecum through sigmoid colon (ICD-O codes 153.0, 153.1, 153.2, 153.3, 153.4, 153.5, 153.6, 153.7), rectosigmoid (ICD-O code 154.0), and rectum (ICD-O code 154.1).

### Tissue samples

Tumour material of colorectal cancer patients was collected after approval by the Ethical Review Board of Maastricht University, PALGA and the NCR. Tissue samples from 819 colorectal cancer patients were localized in 54 pathology laboratories throughout the Netherlands. Forty-four (5%) tumour tissue samples could not be retrieved from the pathology archives. Of 775 available tissue samples, 737 (95%) contained sufficient tumour material for molecular analyses. Tissue sections were cut from each sample, which were used for DNA isolation and immunohistochemical analysis.

### DNA isolation

DNA isolation was described in detail elsewhere [27]. Briefly, a 4 μm section, cut from each paraffin-embedded tumour tissue block, was stained with haematoxylin and eosin (HE) for histopathological examination by a pathologist. Five 20 μm sections of tumour tissue were cut from each sample for DNA isolation. Tumour tissue was separated from normal colon epithelium using the HE section as a reference. Genomic DNA was extracted from macrodissected tumour tissue using proteinase K (Qiagen, St. Louis, MO, USA) and the Puregene DNA isolation kit (Gentra Systems, Minneapolis, MN, USA). DNA concentration and purity was measured at 260 and 280 nm.

### *K-ras *mutation analysis

Mutation analysis of the exon 1 fragment of the *K-ras *oncogene, spanning codons 8–29, was performed on archival colorectal adenocarcinoma specimens of 737 patients, using nested PCR, followed by direct sequencing of purified fragments [27]. The detection limit was 5% mutated DNA and duplicate experiments revealed a good reproducibility (88%) [27].

### *APC *mutation analysis

The majority of somatic mutations in the *APC *gene are found within the mutation cluster region (codons 1286–1520). Mutation analysis of the mutation cluster region was performed on adenocarcinoma DNA using nested PCR for amplification of the mutation cluster region as four overlapping DNA fragments followed by direct sequencing of purified fragments, as previously described [28]. An alternative nested PCR strategy was performed when nested PCR failed for any of the fragments, using different primers. The detection limit was 5% mutated DNA and duplicate experiments revealed good reproducibility (85%)[28]. From 72 of the 737 patients with sufficient DNA yield, one or more fragments of the mutation cluster region could not be amplified and these patients were not included in this study.

### *CTNNB1 *mutation analysis

All 464 samples without a truncating *APC *mutation (n = 411) and all samples with absent hMLH1 expression (n = 58) were analysed for mutations in the phosphorylation sites at codons 33, 37, 41 and 45 in exon 3 of the *CTNNB1 *gene. This selection was made, since most mutations are expected in these samples. Tumours lacking truncating APC mutations may harbour *CTNNB1 *mutations [7], and microsatellite instable tumours are also expected to more frequently have mutations in *CTNNB1 *[26].

Amplification of exon 3 of the *CTNNB1 *gene entailed a semi-nested PCR strategy, which covered codons 33, 37, 41 and 45. Flank PCR was performed to generate a 308 bp fragment (primers, forward: 5'-CCAATCTACTAATGCTAATACTG-3', reverse: 5'-GCATTCTGACTTTCAGTAAGGC-3') that was used in a 1:100 dilution for amplification of the final PCR product (primers, forward: 5'-CCAATCTACTAATGCTAATACTG-3', reverse: 5'-CTTCCTCAGGATTGCCTTTACC-3'). In each PCR, one round of 35 cycles was performed.

The semi-nested PCR products from samples without a truncating *APC *mutation were screened for mutations using denaturing high-pressure liquid chromatography (dHPLC) on a WAVE 3500 HT system (Transgenomic Inc., UK). WAVE analysis was optimised and validated using specific mutations in cell line DNA, i.e. HCT116 (codon 45: 3 bp deletion) and SW48 (codon 33: C→A) as well as DNA derived from desmoid tumours from patients (codon 41: A→G and codon 45: C→T) as positive controls. All of these mutations were repeatedly confirmed by sequencing. WAVE analysis was carried out at two different temperatures (57.7 en 60°C). Samples showing an aberrant elution profile were re-amplified and re-screened. When an aberrant elution profile was confirmed, direct sequencing was performed. All samples without hMLH1 expression were analysed by direct sequencing without screening. The sequence profile was analysed on an ALFexpress II DNA analysis system using ALFwin software (Amersham Biosciences, Roosendaal, the Netherlands).

### hMLH1 expression

Formalin-fixed, paraffin-embedded tissue sections cut at 4 μm, which included tumour tissue with normal adjacent mucosa, were used for immunohistochemistry. Endogenous peroxidase activity was blocked by 3% H_2_O_2_. Slides were submitted to microwave antigen retrieval in 1 mM EDTA buffer (pH 8.0) and incubated with 10% normal horse serum for ten min at room temperature. Then, sections were incubated overnight at 4°C with mouse monoclonal antibodies against hMLH1 protein (clone G168-15, PharMingen, San Diego, CA) at a 1:100 dilution. Antibody binding was detected by incubating the sections at room temperature with the peroxidase-labelled DAKO Envision System (DAKO, Carpinteris, CA), using DAB as a chromogen. Sections were counterstained with diluted haematoxylin.

Lesions were considered to lack hMLH1 protein expression when unequivocal absence of nuclear staining of the tumour epithelial cells was observed. Nuclear staining of normal epithelial and stromal cells or lymphocytes served as an internal positive control. Staining was scored independently by at least two observers and in case of discordant results discussed with a pathologist until consensus was reached. hMLH1 expression could be determined in 724 of 737 patients.

### BAT-26

Analysis of the BAT-26 mononucleotide repeat was performed in a random sample of tumour specimens from 114 patients, and a series of 48 of 58 tumours that lacked hMLH1 expression, to assess the concordance between the microsatellite instability marker BAT-26 and hMLH1 expression. The primer sequences and PCR conditions for the BAT-26 mononucleotide repeat were used as described previously [31].

### Statistical analysis

In the statistical analysis, data from 656 patients for whom information on *APC *and *K-ras *mutation status as well as hMLH1 expression was complete were included.

The χ^2 ^test and Cramérs V test were used to estimate the association of the co-occurrence of *K-ras *and *APC *gene mutations. Characteristics of patients (age at diagnosis, sex, family history of colorectal cancer) and tumours (tumour sub-localisation, Dukes' stage and tumour differentiation) were compared between patients with and without an activating *K-ras *or a truncating *APC *mutation as well as patients harbouring tumours with and without hMLH1 expression, using Students T-test (age at diagnosis) and χ^2 ^tests (sex, family history of colorectal cancer, tumour sub-localisation, Dukes' stage and differentiation). Additionally, patient and tumour characteristics of tumours with an activating *K-ras *and/or a truncating *APC *mutation were compared to tumours lacking hMLH1 expression. All *P*-values are reported for a two-sided test; *P*-values of less than 0.05 were considered to be statistically significant.

## Results

Tumours from 464 of 656 patients, which did not harbour a truncating APC mutation or lacked hMLH1 expression, were analysed for mutations in exon 3 of the *CTNNB1 *gene. Table 1 describes the tumour and patient characteristics of seven colorectal tumours that harboured a mutation in *CTNNB1 *exon 3. In five colorectal cancers, a *CTNNB1 *mutation that would lead to loss of one of the Ser/Thr phosphorylation sites and subsequent stabilisation of the protein, occurred at codons 37 and 45, all were C→T transitions, leading to Ser→Phe amino acid changes and occurred in the proximal colon. All bar one also had an activating mutation in the *K-ras *gene. Three of these five tumours showed hMLH1 deficiency. Two colorectal cancer patients harboured a mutation in the *CTNNB1 *gene, that did not occur at the Ser/Thr phosphorylation sites, but would result in an amino acid alteration at codons 22 and 29, the effects of which are unknown. Because of the very low frequency of tumours harbouring a *CTNNB1 *mutation, these mutations were not included in further analyses. In addition, mutation analysis of remaining samples was abandoned, since this was deemed irrelevant as these harboured truncating *APC *mutations and are considered to be unlikely to also have *CTNNB1 *mutations [7].

**Figure 1:**
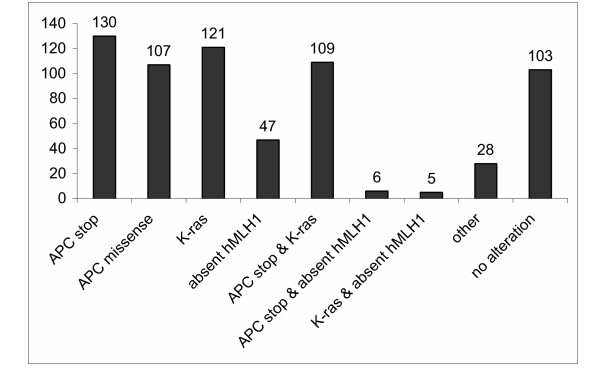
Distribution of aberrations in the *APC *and *K-ras *genes and hMLH1 expression in 656 colorectal tumours. The data in this figure are based on tumours from 656 sporadic colorectal cancer patients. The bars representing the different molecular alterations represent mutually exclusive groups of tumours. The category "Other" includes; *APC *silent mutation (n = 25), *K-ras *mutations other than missense mutations in codons 12 and 13 (n = 2), and both of these (n = 1).

Of 656 tumours for which the other molecular alterations, i.e. mutations in the *APC *and *K-ras *genes and hMLH1 expression, were all successfully and completely analysed, 103 colorectal tumours did not harbour a truncating or missense *APC *mutation, an activating *K-ras *mutation or showed lack of hMLH1 expression, as depicted in figure 1. Truncating as well as missense *APC *mutations and activating *K-ras *mutations were relatively common. Truncating *APC *mutations alone and activating *K-ras *mutations in codons 12 and 13 only, occurred at similar frequencies (20% (130/656) and 18% (121/656), respectively). A combination of a truncating mutation in *APC *and an activating mutation in *K-ras *occurred less often than the sole occurrences of mutations in both genes. However, as shown in table 2, the simultaneous occurrence of mutations in both genes occurred more frequently than expected on the basis of chance alone. A χ^2 ^test for the occurrence of a truncating *APC *mutation and an activating *K-ras *mutation revealed that the occurrence of these mutations was not independent (χ^2 ^= 8.7, *P *< 0.001), but the correlation was weak (Cramérs V = 0.138). Finally, although 11 tumours that harboured a mutation in the *APC *or *K-ras *gene also lacked hMLH1 expression, hMLH1 deficiency occurred more frequently in tumours that did not harbour these mutations (χ^2 ^= 36.6, *P *< 0.001).

**Figure 2:**
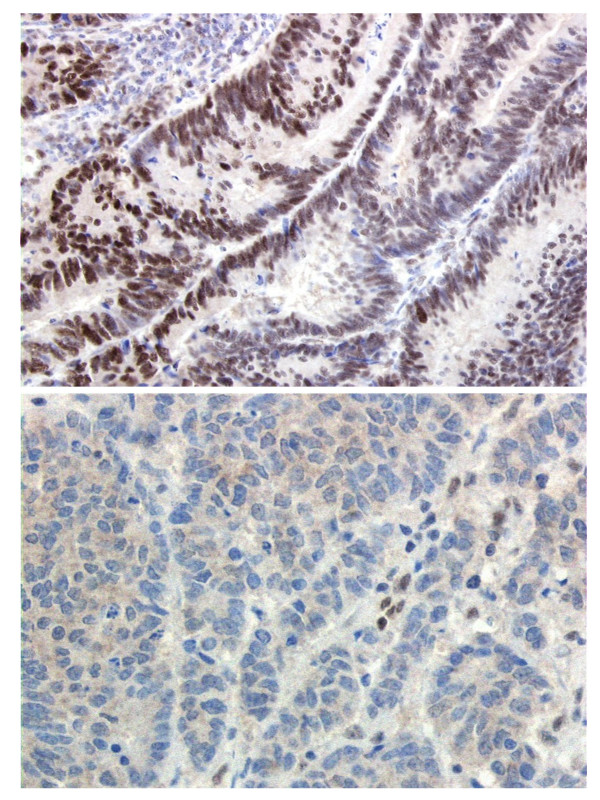
hMLH1 immunohistochemistry. Present (top panel) and absent (bottom panel) immunohistochemical staining for hMLH1.

With respect to the localisation in the colorectal tract, tumours of the rectosigmoid and rectum more frequently harboured truncating *APC *mutations when compared to colon tumours (*P *= 0.001), as shown in table 3. Rectosigmoid and rectal tumours have a relatively higher frequency of *K-ras *mutations in codons 12 and 13 when compared to colon tumours (*P *= 0.05) (Table 3). Nine per cent of tumours showed hMLH1 deficiency, as determined by immunohistochemistry (Figure 2). Tumours lacking hMLH1 expression occur almost exclusively in the proximal colon (*P *< 0.001) and relatively more frequently show poor differentiation or are undifferentiated (*P *< 0.001) when compared to tumours with hMLH1 expression (Table 3).

Next, we compared the patient and tumour characteristics of tumours harbouring a truncating *APC *and/or an activating *K-ras *mutation to those of tumours without hMLH1 expression, and these results are presented in table 4. Patients harbouring hMLH1 deficient tumours were slightly older when diagnosed with colorectal cancer (69.3 yr (68.0–70.5) versus 67.8 (67.4–68.3), *P *= 0.03), were relatively less frequently men (40% versus 58%, *P *= 0.02). Tumours without hMLH1 expression occurred relatively more frequently in the proximal colon (*P *< 0.001) and relatively more frequently showed poor differentiation or are undifferentiated (*P *< 0.001).

When comparing tumours with a missense (but not a truncating) mutation in *APC *to tumours with a truncating mutation in *APC*, missense mutations occurred relatively more frequently in the colon (*P *= 0.002), less often also harboured an activating *K-ras *mutation (*P *= 0.004), and more often also lacked hMLH1 expression (*P *< 0.001). No differences were observed with regard to age at diagnosis, gender, Dukes' stage or tumour differentiation (data not shown).

Finally, to assess agreement between hMLH1 expression and microsatellite instability, both hMLH1 expression and BAT-26 were analysed in 162 tumours. All tumours that had normal BAT-26, also showed hMLH1 expression. Fourteen tumours with unstable BAT-26 also lacked hMLH1 expression, and two tumours with unstable BAT-26 were found to express hMLH1, which demonstrates a high agreement between these molecular features of mismatch repair deficiency.

## Discussion

In this study, the occurrence of mutations in the *APC*, *CTNNB1 *and *K-ras *genes as well as expression of the hMLH1 protein in tumour tissue of 656 sporadic colorectal cancer cases were investigated. The occurrence of mutations in the *CTNNB1 *gene, which codes for β-catenin, was rare: only five of 464 tumours analysed were found to have a mutation at one of the phosphorylation sites in exon 3. Truncating mutations in *APC *and activating mutations in *K-ras *appeared to occur at similar frequencies. Although tumours harbouring both mutations were relatively rare, mutations in *APC *and *K-ras *seemed to occur co-dependently. Nine percent of all tumours (58/656) lacked hMLH1 expression, and in these tumours almost no *APC *or *K-ras *mutations was detected. Patients harbouring a tumour with absent hMLH1 expression were older, more often women, more often had proximal colon tumours that showed poorer differentiation when compared to patients who harboured a tumour with an *APC *and/or *K-ras *mutation.

The selection of patients included in this study was based on the completeness of analyses of both *APC *and *K-ras *genes as well as hMLH1 expression and this led to a considerable reduction in the number of cases that could be included in the analyses presented in this study. The largest reduction (72 cases) was due to incompleteness of the analysis of all fragments comprising the *APC *mutation cluster region. Tumour DNA was derived from formalin-fixed, paraffin-embedded tumour tissue blocks. Depending on the conditions of fixation and storage, the extracted DNA is more or less fragmented, which may have impaired the analysis of mutations in the *APC *gene more than in the *K-ras *gene, since the analysis of the latter is based on the amplification of a smaller gene fragment. It should be emphasized that characteristics of patients (age, sex, family history of colorectal cancer) and tumours (sub-localisation, Dukes' stage and differentiation) of the group under study are similar to the 737 patients for whom tumour material was available and to all 819 patients initially recognized within the cohort (data not shown). Moreover, the *K-ras *and hMLH1data presented here are similar to the data for *K-ras *and hMLH1 based on the complete groups (737 and 724 cases, respectively) (data not shown).

Mutations in exon 3 of the *CTNNB1 *gene leading to loss of one of the phosphorylation sites were rare. Strikingly, all five of these mutations occurred in the proximal colon and three of these also had absent hMLH1 expression. This may indicate that these proximal colon tumours, which often also show mismatch repair deficiency, are more likely to harbour *CTNNB1 *mutations. This was also found in a study of microsatellite instable colorectal tumours [26]. The WAVE screening technique has not been used previously for formalin-fixed, paraffin-embedded tissue, and therefore it seems plausible that samples harbouring a *CTNNB1 *mutation have escaped detection. However, all 58 hMLH1 deficient samples were analysed by direct sequencing without a prior screening step, and only three of these samples harboured a *CTNNB1 *mutation, indicating the low frequency of such mutations.

The model describing the accumulation of genetic alterations of the *APC*, *K-ras*, *TP53 *and *SMAD4 *genes that drive the development of a carcinoma, has become generally accepted as a paradigm for the genetic basis of colorectal carcinogenesis [3,32]. The relatively low frequency of simultaneous occurrence of mutations in both *APC *and *K-ras *observed in this study seems to argue against this synergy. This contention is in accordance with observations from another cohort study, in which *APC*, *K-ras *and *TP53 *gene mutations were studied in 109 tumours and these mutations were found to rarely occur together in the same tumour [9]. However, the simultaneous occurrence of *APC *and *K-ras *mutations observed in our study occurs more frequently than expected based on chance alone and therefore mutations in the *APC *and *K-ras *genes do not seem to occur independently. When data on *APC *and *K-ras *mutations are derived from the study by Smith *et al*. [9], similar results, although not statistically significant, could be obtained.

The *K-ras *mutation frequency of 37% is in accordance with reported frequencies of 30 to 60% [33-43]. The frequency of 37% of truncating mutations in the mutation cluster region of *APC *in this study, however, seems low in comparison to the general assumption that most colorectal tumours harbour a mutation in the *APC *gene. When only reports from studies on sporadic rather than familial colorectal cancer or colorectal cancer cell lines are considered, the mutation frequencies are lower and vary between 30 and 70% [17,44-49], and a population-based case-control study in the Netherlands reported a 32% mutation frequency [50].

The method for mutation analysis of the *APC *mutation cluster region and exon 1 of *K-ras *is based on nested amplification and direct sequencing of purified PCR fragments, a highly sensitive method. Since no screening step was performed prior to the sequencing of the gene fragments, it is unlikely that mutations would have escaped detection. The reproducibility of the applied assays was good, with a reproducibility of 85% and 88% for *APC *and *K-ras*, respectively. Arguably, this indicates the extent of heterogeneity present in the tumour samples.

In 103 sporadic colorectal cancers no alterations were found in the *K-ras*, *APC *or hMLH1 genes. It is plausible that these tumours have harboured mutations in other components of the Wnt signalling pathway, e.g. mutations in the Axin genes, which are essential for the degradation of β-catenin, and were observed in 11% of patient samples [51]. In addition, an epi-genetic change, i.e. promotor hypermethylation of the *APC *gene that leads to impaired APC function has been observed in 18% of sporadic colorectal adenomas and carcinomas [52].

Of the microsatellite instable tumours, approximately 90% show absence of hMLH1 expression [24]. In most sporadic colorectal cancers, the promoter region of hMLH1 is hypermethylated, resulting in absence of the protein [53-55]. BAT-26 was previously shown to identify microsatellite instability in sporadic colorectal cancer [56] and results from our study showed a good agreement between unstable BAT-26 and absent hMLH1 expression.

Several studies have shown that microsatellite instability and mutations in *APC *and *K-ras *occur almost mutually exclusively [18,19,21,57], suggesting that these characteristics represent separate pathways. However, others have observed aberrations in both pathways, but these studies have been performed in relatively small groups of HNPCC and sporadic colorectal cancer patients [14,20,58] and this may have given rise to a relative overrepresentation of mutation detection in both pathways. Although in our study the simultaneous occurrence of hMLH1 deficiency as well as an *APC *or *K-ras *mutation was observed in a small number of tumours, the mutually exclusive occurrence of hMLH1 deficiency and mutations in *APC *and/or *K-ras *seemed to predominate.

Moreover, after exclusion of tumours displaying this overlap, a striking difference between occurrences of *APC *and/or *K-ras *mutations versus absence of hMLH1 expression was observed. The differences were most pronounced with regard to tumour sub-localisation and differentiation. hMLH1 deficient tumours occur almost exclusively in the proximal colon and are relatively more frequently poorly differentiated, which is in accordance with reports from other studies [22-25].

## Conclusion

In conclusion, this study shows that mutations in the *CTNNB1 *gene are presumably of minor importance in sporadic colorectal cancer. Although *APC *and *K-ras *mutations are often observed seperately in a tumour, these mutations seem to also occur in a co-dependent manner. Tumours that display mismatch repair deficiency, may form a distinct sub-group as they differ from tumours with *APC *and/or *K-ras *mutations with regard to age, sex, tumour sub-localisation and differentiation.

## List of abbreviations used

APC: adenomatous polyposis coli

TP53: tumour protein 53

K-ras: Kirsten ras

hMLH1: human mut-L homologue 1

NLCS: Netherlands Cohort Study on diet and cancer

NCR: Netherlands Cancer Registry

PALGA: Pathologisch Anatomisch Landelijk Geautomatiseerd Archief

HE: haematoxylin and eosin

## Competing interests

The authors declare that they have no competing interests.

## Authors' contributions

ML participated in the study design, carried out the statistical analysis, interpreted the data, and drafted the manuscript. MPW conceived the study, and participated in its design and coordination, and revised the manuscript. PAW carried out the immunohistochemistry of hMLH1 and critically reviewed the manuscript. AMS carried out the β-catenin analysis. GMJMR carried out DNA isolation and the *APC *and *K-ras *analyses. GNPvM participated in the study coordination and critically reviewed the manuscript. APdB evaluated the tissue samples for tumour content and critically reviewed the manuscript. PAvdB conceived the study, and participated in its design and coordination and critically reviewed the manuscript. AFPMdG conceived the study, and participated in its design and coordination, and revised the manuscript. All of the authors have read and approved the final manuscript.

## Pre-publication history

The pre-publication history for this paper can be accessed here:



## Figures and Tables

**Table 1:** Description of patient, tumour and molecular characteristics of colorectal cancers harbouring a *CTNNB1 *mutation.

Patient^a^	Sex	Age at diagnosis (yr)	Sub-localisation	Dukes' stage	Tumour differentiation	Codon	Mutation	Amino acid change	hMLH1 expression^b^	Truncating *APC *mutation^b^	Activating *K-ras *mutation
1	M	65	Proximal colon	B	Moderate	37	C→T	Ser→Phe	Present	No	Yes
2	M	67	Proximal colon	A	Moderate	45	C→T	Ser→Phe	Absent	No	Yes
3	M	69	Proximal colon	B	Good	45	C→T	Ser→Phe	Absent	No	No
4	M	61	Proximal colon	C	Moderate	45	C→T	Ser→Phe	Present	No	Yes
5	F	69	Proximal colon	A	Undifferentiated	45	C→T	Ser→Phe	Absent	No	Yes
6^c^	M	69	Distal colon	B	Moderate	22	G→A	Val→Ile	Present	No	Yes
7^c^	F	65	Proximal colon	A	Moderate	29	C→A	Ser→Tyr	Present	No	No

^a ^Tumours of 464 sporadic colorectal cancer patients were analysed.

^b ^58 Tumours were selected based on absence of hMLH1 expression, 411 tumours studied were selected based on absence of a truncating *APC *mutation.

^c ^Two patients (numbers 6 and 7) harboured a *CTNNB1 *mutation that would not lead to loss of a known phosphorylation site.

**Table 2:** Crosstabulation of patients with and without activating *K-ras *and truncating *APC *mutation(s).

Activating K-ras mutation	Truncating APC mutation		
	No	Yes	Total
No	258	136	421
expected^a^	263.8	157.2	
Yes	126	109	235
expected^a^	147.2	87.8	
Total	411	245	656

^a ^Expected based on chance alone. χ^2 ^= 8.7, *P *< 0.001

**Table 3:** Comparisons between patients with or without a *K-ras *or *APC *mutation and hMLH1 expression.

	Genetic aberration											
	*APC*^a^				*K-ras*^b^				hMLH1 expression			

	No mutation (truncation)		Mutation (truncation)		No mutation (codon 12/13)		Mutation (codon 12/13)		Present		Absent	

Number of patients (%)	411	(63)	245	(37)	421	(64)	235	(36)	598	(91)	58	(9)
Age at diagnosis (yr, mean (SD))	68.2	(4.3)	67.5	(4.2)	67.7	(4.3)	68.3	(4.3)	67.9	(4.2)	68.7	(4.7)
*P-value*	*0.07*				*0.08*				*0.18*			
Sex (% male)	54		58		56		55		57		45	
*P-value*	*0.45*				*0.84*				*0.08*			
Family history of colorectal cancer (% yes)	10		9		11		7		10		9	
*P-value*	*0.89*				*0.07*				*0.79*			
Tumour sub-localisation^c^												
Proximal colon	151	(69%)	67	(31%)	141	(65%)	77	(35%)	169	(78%)	49	(12%)
Distal colon	138	(68%)	66	(32%)	144	(71%)	60	(29%)	199	(98%)	5	(2%)
Rectosigmoid	34	(47%)	39	(53%)	44	(60%)	29	(40%)	73	(100%)	0	(0%)
Rectum	83	(55%)	67	(45%)	85	(57%)	65	(43%)	146	(97%)	4	(3%)
*P-value*	*<0.001*				*0.05*				*<0.001*			
Dukes' stage^c^												
A	94	(60%)	63	(40%)	103	(66%)	54	(34%)	142	(90%)	15	(10%)
B	147	(67%)	73	(33%)	142	(65%)	78	(35%)	192	(87%)	28	(13%)
C	97	(60%)	64	(40%)	107	(66%)	54	(34%)	151	(94%)	10	(6%)
D	39	(54%)	33	(46%)	40	(56%)	32	(44%)	69	(96%)	3	(4%)
*P-value*	*0.21*				*0.42*				*0.07*			
Differentiation^c^												
Good	35	(56%)	28	(44%)	40	(63%)	23	(37%)	59	(94%)	4	(6%)
Moderate	250	(62%)	156	(38%)	258	(64%)	148	(34%)	380	(94%)	26	(6%)
Poor	66	(69%)	30	(31%)	68	(71%)	28	(29%)	82	(85%)	14	(15%)
Undifferentiated	6	(86%)	1	(14%)	5	(71%)	2	(29%)	4	(57%)	3	(43%)
*P-value*	*0.20*				*0.57*				*<0.001*			

^a ^*APC *mutations: only mutations resulting in a stop codon.

^b ^*K-ras *mutations: only activating mutations in codon 12 or 13.

^c ^Tumour sublocalisation, Dukes's stage and differentiation gave rise to missing values.

**Table 4:** Comparisons between patients with an *APC *and/or *K-ras *mutation and absent hMLH1 expression.

	*APC*^b ^and/or *K-ras*^c ^mutation		Absence of hMLH1		*P*-value
Number of patients^a^	360		47		
Age at diagnosis (yr, mean (SD))	67.8	(4.2)	69.3	(4.3)	0.03
Sex (% male)	58		40		0.02
Family history of colorectal cancer (% yes)	8		11		0.60
Tumour sub-localisation					<0.001
Proximal colon	96	(70%)	41	(30%)	
Distal colon	107	(97%)	3	(3%)	
Rectosigmoid	51	(100%)	0	(0%)	
Rectum	100	(97%)	3	(3%)	
Dukes' stage					0.08
A	86	(90%)	10	(10%)	
B	117	(84%)	23	(16%)	
C	89	(91%)	9	(9%)	
D	47	(94%)	3	(6%)	
Differentiation					<0.001
Good	37	(92%)	3	(8%)	
Moderate	232	(92%)	20	(8%)	
Poor	43	(78%)	12	(22%)	
Undifferentiated	1	(33%)	2	(67%)	

^a ^Patients with both absent hMLH1 expression and an *APC *or *K-ras *mutation were excluded from the analysis.

^b ^*APC *mutations: only mutations resulting in a stop codon.

^c ^*K-ras *mutations: only activating mutations in codon 12 or 13.
